# Pathogenicity and Its Implications in Taxonomy: The *Brucella* and *Ochrobactrum* Case

**DOI:** 10.3390/pathogens11030377

**Published:** 2022-03-21

**Authors:** Edgardo Moreno, José María Blasco, Jean Jacques Letesson, Jean Pierre Gorvel, Ignacio Moriyón

**Affiliations:** 1 Escuela de Medicina Veterinaria, Universidad Nacional, Heredia 40101, Costa Rica; 2 Centro de Investigación y Tecnología Agroalimentaria, Instituto Agroalimentario de Aragón, Universidad de Zaragoza, 50059 Zaragoza, Spain; jblasco@unizar.es; 3 Unité de Recherche en Biologie des Microorganismes, Faculty of Science, University of Namur, 5000 Namur, Belgium; jean-jacques.letesson@unamur.be; 4 Centre d’Immunologie de Marseille-Luminy, Aix-Marseille Université, CNRS, INSERM, CIML, 13009 Marseille, France; 5 Instituto de Salud Tropical y Departamento de Microbiología y Parasitología, Universidad de Navarra, 31008 Pamplona, Spain; imoriyon@unav.es

**Keywords:** *Brucella*, *Ochrobactrum*, brucellosis, genus, species, pangenome, core genome

## Abstract

The intracellular pathogens of the genus *Brucella* are phylogenetically close to *Ochrobactrum*, a diverse group of free-living bacteria with a few species occasionally infecting medically compromised patients. A group of taxonomists recently included all *Ochrobactrum* organisms in the genus *Brucella* based on global genome analyses and alleged equivalences with genera such as *Mycobacterium*. Here, we demonstrate that such equivalencies are incorrect because they overlook the complexities of pathogenicity. By summarizing *Brucella* and *Ochrobactrum* divergences in lifestyle, structure, physiology, population, closed versus open pangenomes, genomic traits, and pathogenicity, we show that when they are adequately understood, they are highly relevant in taxonomy and not unidimensional quantitative characters. Thus, the *Ochrobactrum* and *Brucella* differences are not limited to their assignments to different “risk-groups”, a biologically (and hence, taxonomically) oversimplified description that, moreover, does not support ignoring the *nomen periculosum* rule, as proposed. Since the epidemiology, prophylaxis, diagnosis, and treatment are thoroughly unrelated, merging free-living *Ochrobactrum* organisms with highly pathogenic *Brucella* organisms brings evident risks for veterinarians, medical doctors, and public health authorities who confront brucellosis, a significant zoonosis worldwide. Therefore, from taxonomical and practical standpoints, the *Brucella* and *Ochrobactrum* genera must be maintained apart. Consequently, we urge researchers, culture collections, and databases to keep their canonical nomenclature.

## 1. Introduction

The *Brucella* organisms are among the first recognized zoonotic bacteria, becoming classic examples in the history of infectious diseases. However, since these bacteria do not show conspicuous traits upon culture and because the disease they cause (brucellosis) lacks pathognomonic signs and symptoms, it has taken over 100 years to realize the close resemblance of strains isolated from different hosts and to define the *Brucella* genus; investigation in which the peculiar pathogenicity and epidemiology of these bacteria were key [1]. Taxonomically, minimum standards for the *Brucella* genus were defined in 1975 emphasizing virulence and intracellular pathogenicity as required traits for including species in this genus [2]. About a decade later, a group of investigators showed that these pathogens were close to some soil bacteria that occasionally caused opportunistic nosocomial infections, known as the CDC group Vd or *Alcaligenes*/*Achromobacter* cluster [3,4,5], subsequently described as the *Ochrobactrum* genus [6]. It soon became evident that the *Brucella* and *Ochrobactrum* organisms belong to the Class 2 Alphaproteobacteria, thus closely related to plant pathogens and endosymbionts such as *Agrobacterium* and *Sinorhizobium* species [4,5,7]. Therefore, both genera are part of the so-called Rhizobiales, from the taxonomical perspective within *Brucellaceae*, a sister group of *Bartonellaceae* [8]. From the beginning, the *Brucella* and *Ochrobactrum* species have been maintained in two different genera based on relevant phenotypic, genotypic, biological, and epidemiological differences. A group of taxonomists recently joined these two groups in a single genus, “*Brucella*”, based on a BLAST Distance Phylogeny approach (a cladistic methodology that uses only genes identified as orthologs using bioinformatic tools) and a supposed equivalence with some genera of pathogenic bacteria [9]. The arguments put forward to justify this claim follow (for simplicity, we omitted the citations):

“The overall genomic divergence of the *Brucella*-*Ochrobactrum* clade was lower than in many clades harboring a single genus only. *Brucella* differs from *Ochrobactrum* regarding its pathogenic lifestyle, which may be reflected in the smaller genome size of *Brucella*. However, *Ochrobactrum* species are also known from clinical specimens, including its type species, and a more pronounced genome size reduction of pathogenic species nested within a partially non-pathogenic genus was observed elsewhere, as, e.g., in *Mycobacterium leprae*. *Mycobacterium* can also serve as an example for a genus harboring distinct risk groups, much like *Burkholderia* and *Yersinia*. Hence, the difference between *Brucella* and *Ochrobactrum* regarding their risk-group assignment could hardly be used as an argument against their inclusion in the same genus. Known phenotypic differences, if any, appeared to be restricted to autapomorphies of *Brucella* that may well be linked to its evolutionary adaptation to pathogenesis. Despite the differences in genome size, the gene-content analysis provided more support for the combined *Brucella*–*Ochrobactrum* clade than for the subclades”.

[9] (Reproduced under Creative Commons CC-BY license)

Here, we argue that including *Ochrobactrum* in the genus *Brucella* following such genomic analysis and supposed equivalences with other genera of pathogens is incorrect. After briefly reviewing the different criteria used by biologists to delineate the genus, we analyze the *Ochrobactrum* versus *Brucella* case and show that a genus concept that emphasizes cladistics disregarding typology (i.e., in-depth genomic analysis, structure and physiology, population structure, ecology, and lifestyle) is insufficient, a deficiency particularly clear in the case of pathogens. We conclude that a correct taxonomical approach needs to include a proper understanding of pathogenicity. Moreover, concerning the highly infectious and vicious *Brucella* organisms, we argue that any taxonomical approach requires the multiple dimensions of pathogenicity and the practical implications of virulence. Consequently, we concluded that *Ochrobactrum* and *Brucella* organisms must be maintained apart in two different genera.

## 2. The Concept of Genus

After Linnaeus, biologists used a binomial nomenclature to which the name for an organism was based on two terms, the genus and the species, and this naming convention has been maintained in modern taxonomy. While there have been numerous debates regarding the species concept in sexual and asexual organisms, the genus and other higher taxonomical taxa have received less attention because they are much more elusive than the species [10], the entities active in nature. Since the genus and other higher taxonomical hierarchies result from averaging and factorizing common characteristics of known extinct and extant species within a group, they are undoubtedly artificial constructs of the human mind intended to bring a practical order in our observations of biological diversity [11,12]. Accordingly, the taxonomic effort is not, *sensu stricto*, a scientific endeavor but an epistemological one that includes two dialectically inseparable procedures: the analytical and the typological processes.

### 2.1. The Analytical Process

The analytical process uses quantitatively measurable characteristics of the subjects organized in ranks, commonly depicted as cladograms or phylogenetic trees, which are hypotheses on the evolutionary course of the species. It must be borne in mind that, whereas this analysis is of fundamental importance in evolutionary studies, these representations are based on numerical values and thus, depend on the parameters quantified. Not surprisingly, in genomic sequence comparisons, the distance between two groups with different genome sizes (the case of *Ochrobactrum* and *Brucella*, see below) may be enlarged or narrowed depending on whether the tree is constructed based on SNPs, core genomes, pangenomes, or nonsynonymous or synonymous sites, among several options [13]. Nonetheless, the punctuated ancestor-descendant relationships depicted through dichotomic nodes and branches in a tree are representations based on similarities presented as numerical values. Still, the interpretation of these values follows circular reasoning (the principle of *circulus in probando*) because a group of organisms descended from a common ancestor (premise) are closely related (conclusion), and they are closely related (premise) because they descended from a common ancestor (conclusion). In addition, drawing a cut-off line in a given branch for defining a cluster of organisms and including them in a taxon, such as a genus, is a decision that cannot be taken solely on quantitative parameters and thus, is usually based on assessments made by other methods [14,15,16]. Moreover, the branching points are not fixed features of the possible trees because we do not know the characteristics of the still undiscovered or extinct individuals not incorporated in the analysis. Consequently, the clustering or the division of groups (including the genus) reflects perspectives that, although obtained after analyzing multiple characteristics, are thus strictly speaking anthropocentric.

### 2.2. The Typological Process

The typological process follows a methodology that conceptually separates items in various dimensions by exploring common and exclusive properties to identify an “ideal type.” Therefore, this process is an intellectual construction that stresses specific properties of different realities that, although critically important, are not necessarily linked to quantitative characters (as those used in cladistics). Thus, the typological process is a flexible heuristic system that adapts to decision-making while working with complex data [17]. Typologies are crucial because they provide efficient and practical bases for comparisons and a framework for giving operative and predictive names, two essential conditions of taxonomy [12] that are particularly relevant in the case of pathogenic bacteria.

Ideally, the analytical and typological essences coincide in a dialectic relationship. However, while acquiring the former (e.g., DNA sequence comparisons) is a straightforward two-dimensional process often amenable to quantification, the typological essence (e.g., ecology, lifestyle, pathogenicity, etc.) includes more than two dimensions and, therefore, requires qualitative assessments. For example, the biological species concept of the famous evolutionist Ernst Mayr (1904–2005) stated that, within a defined cluster (through the analytical process) of living organisms with common characteristics, species are those “groups of actually or potentially interbreeding natural populations, which are reproductively isolated from other such groups” [18]. This famous Mayr’s declaration, mainly used to define most species throughout the plant and animal kingdoms, stressed the typological essence of reproduction as the epistemological conceptualization of the species as a taxon on which evolution occurred through gene flow by interacting phenotypes. The typological characteristics are even more relevant when defining two different genera since they display different “types” precluding reproduction. As expected, in other live systems such as the prokaryotes, where conjugation can be entirely precluded or occur between the same species, between different species, or among species of different genera, the typological principles are more difficult to grasp; however, they cannot be ignored because they are at the core of the diversity of life that taxonomy aims to represent.

### 2.3. The Analytical and Typological Processes Define the Genus

The integration of the analytical and typological processes is reflected in different proportions in the two main ways biologists interpret the genus, i.e., cladistics and evolutionary systematics [19]. The cladistic definition relies heavily or solely on the quantitative analytical process and proposes that a genus is a group of species more closely related among them than with the species of another genus, implying that it must be monophyletic. However, this definition makes no provision that paraphyletic subsets in a monophyletic group can be diverging evolutionary units occupying widely different ecological niches. Furthermore, as discussed above, defining monophyly using quantitative approaches (i.e., strict cladistics) is not straightforward and cannot avoid subjective considerations. A 94.5% or lower sequence identity for two 16S rRNA genes has been proposed as evidence for distinct genera. However, this represents a practical convention to introduce order in the taxonomy of uncultured archaea and bacteria, not a taxonomically objective truth [16]. In fact, the authors of this proposal explicitly acknowledge that such a threshold is a minimum value that does not preclude the formation of separate genera with higher sequence identities if supported by other phenotypic, genetic, or environmental data (the case of *Ochrobactrum* and *Brucella*, see below) [16].

Moreover, it has been emphasized that such a “lower cut-off window” of 16S rRNA gene sequence similarity, while reasonable for the above-cited practical purpose, was based on the evaluation of genera previously delineated by a broad spectrum of methods [15,16]. Clearly, these cautions in applying “lower cut-off windows” illustrate why integrating the analytical and typological process is necessary and support the second interpretation of the genus, i.e., the systematic evolutionary definition. This definition postulates that a genus is a group of species of common ancestry (or a single species for monospecific genera) that occupies an ecological situation different from that occupied by the species of another genus. Following this definition, the genus can be monophyletic or paraphyletic (when the latter is a subset of a monophyletic group), thus, bypassing the problem of strictly defining the branching nodes that occurs in cladistics. Emphasizing ecology (and, therefore, its structural and physiological bases) also introduces typology and thus, becomes operative and predictive. The need to incorporate ecology in taxonomy is obvious, and it is entirely relevant in the case of those bacteria (commensal or pathogens) that colonize and thrive in a given host. In addition, the systematic evolutionary definition considers the evolutionary hypotheses derived from cladistic analyses and puts them in a biologically and taxonomically meaningful perspective. Specifically, in the context of our discussion of *Brucella* and *Ochrobactrum*, it is necessary to consider hypotheses on how bacterial pathogens emerge and evolve in nature.

## 3. Pathogenicity and Its Taxonomical Implications: The *Brucella* and *Ochrobactrum* Case

Below, we discuss why the equivalence arguments proposed by Hördt et al. [9] to merge *Ochrobactrum* and *Brucella* in a single genus are incorrect and why, when the analytical and typological approaches are appropriately applied, the *Brucella* and the *Ochrobactrum* organisms are clearly identified as widely different groups each belonging to a different genus. Their overall characteristics are summarized in Table 1 and used in the following discussions.

### 3.1. Pathogenicity and False Equivalence Arguments

Assuming that the *Brucella* and *Ochrobactrum* species should be part of the same genus because other investigators clustered bacteria with higher genomic divergence within the same genus follows what, in logic, is called a false equivalence structure of propositions. This case partly follows from the misapplication of the Taxonomic Principle of Balance. This taxonomy principle states that “retrieval of information is greatly facilitated if the taxa at a given categorical rank are, as far as possible, of equal size and degree of diversity” [64], which is, in any case, based on practical anthropocentric reasons, and not explicitly and accurately aimed to describe what exists in nature (the bold text shows why this principle does not support merging *Ochrobactrum* and *Brucella* because these genera show widely different internal diversity). Apparent equivalences are not uncommon since two things that are alike in some aspects are not necessarily alike in other aspects, as illustrated by many examples in taxonomy. One of the best-known is the *Shigella* and *Escherichia* dichotomy. Even though *Shigella* and *Escherichia* strains are phylogenetically entwined, they are maintained apart because of significant pangenomic differences, including chromosomal sizes, insertion sequence-mediated pseudogenization, acquisition of the pINV virulence plasmid, and above all, different typologies [32,65]. The problem is evident when the *Mycobacterium* cluster (the equivalence example given in [9]) is examined in detail and compared with the *Ochrobactrum*-*Brucella* case. The diagnosis, medical care, and recommended antibiotic treatment are mostly the same regardless of whether nontuberculous or tuberculous mycobacteria cause the infection. The diagnosis of lung disease caused by nontuberculous or tuberculous bacilli requires the integration of clinical, radiographic, and microbiological information and the isolation of the microorganisms from sputum by bronchoscopic lavage or biopsy with granulomatous inflammation histopathological features, and the presence of acid-fast bacilli, no matter the mycobacterial strain, and the disease overall known as tuberculosis [66]. Therefore, symptomatic patients with compatible radiographic findings should meet the microbiological criteria to diagnose mycobacterial lung disease, in all cases taking the same precautions for the appearance of antibiotic-resistant strains [67]. All these characteristics of mycobacterial infections reflect a common biological pattern that does not exist between the *Brucella* and *Ochrobactrum* organisms because the respective clinical picture, pathogenesis, virulence, infection strategies, diagnosis, treatment, lifestyle, epidemiology, and health impacts are utterly unrelated and even opposed (see below). Indeed, *Mycobacterium*, *Ochrobactrum*, and *Brucella* organisms have evolved in different ecological niches and under different selection forces. Thus, using the mycobacterial group (or bacteria such as *Yersinia* and *Burkholderia*) as an analogy to support joining *Brucella* and *Ochrobactrum* in a single genus is an oversimplification; as such, it has no actual taxonomical value. On the contrary, if something can be learned from these comparisons, it is that each group of living organisms must be analyzed within its own evolutionary context and from a biological, epidemiological, medical, and historical perspective.

### 3.2. Cladistics, Genome Comparisons, and Pangenomes

The limitations of a genus definition that considers primarily or only cladistic arguments have been underlined above, and they become evident when considering the *Ochrobactrum* and *Brucella* case.

**Figure 1:**
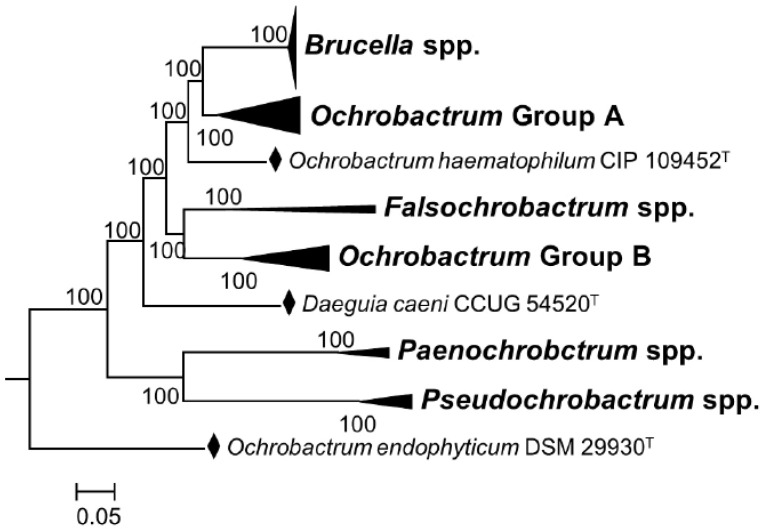
Phylogenetic relationships among genera of the family *Brucellaceae* based on whole sequence genome analysis (a black diamond denotes type strains). Node labels give percentage bootstrap support. *Rhizobium etli* (CFN42T) was used to root the phylogenetic tree (not shown). The tree was constructed using the maximum likelihood method, based on the general time-reversible model, as described by Ashford et al. [30] (adapted from Figure 4 of this reference).

It is true that through cladistics, the members of the genus *Ochrobactrum* (particularly *O. anthropi* and *O. intermedium*) are the closest known relatives of *Brucella* organisms. However, the merit of these studies is that they are helpful to understand the widely diverging (and thus, taxonomically relevant) adaptive evolution of these bacteria, including taxonomically significant examples of exaptation (see below). However, even from a cladistic perspective, the classical and non-classical *Brucella* species constitute a monophyletic group that branches in a distinct and conspicuous clade from other members of the *Brucellaceae*, including the *Ochrobactrum* species [8,30]. When the analyses are focused on core genes identified in the complete genomes using bioinformatic tools, the distinctive clustering of the genus *Brucella* becomes evident, in contrast to the polyphyletic nature of the genus *Ochrobactrum* [8,30] (Figure 1). These differences are overlooked in the analyses allegedly supporting the inclusion of the latter genus in the former one [9]. They are also mainly quantitative and, thus, do not represent the profound biological implications of the genomic differences and clear-cut autapomorphies.

**Figure 2:**
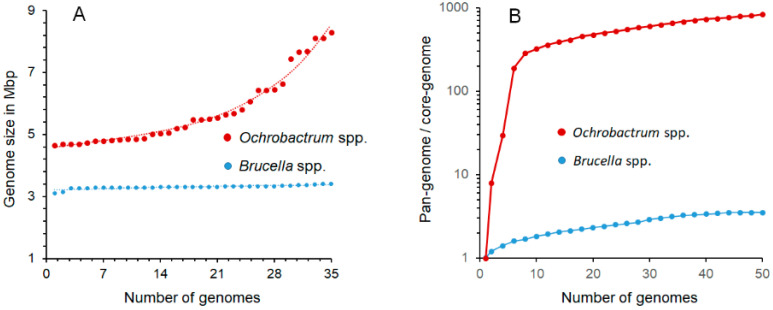
Comparisons of the genome and pangenome sizes of *Ochrobactrum* and *Brucella* organisms. (**A**) While the smaller *Brucella* genomes display a narrow range of sizes, the larger *Ochrobactrum* genomes show significant size variations across the genus; (**B**) the pronounced slope of the curve (shown in logarithmic scale) with a positive trend of *Ochrobactrum* pangenome/core genome ratio indicates a widely open pangenome, in which the complete gene repertoire cannot be predicted with confidence, since the diversity of strains with additional genes keeps increasing due to the continuous shuffling of genes across the species. In contrast, the shallow slope of the pangenome/core genome ratio curve strongly suggests a close pangenome with no further horizontal gene exchange in the extant *Brucella* species. The prediction is that as more *Brucella* genomes are included, the numbers of conserved and accessory genes would remain, with just a limited number of additions and a proportion of ~3.6 unique-accessory genes per core conserved gene. Accordingly, the overall *Brucella* coding genome repertoire of the *Brucella* pangenome can be predicted, with some accuracy, in ~11,000 genes. Data to construct the graphs were retrieved from [20,22,24,25,27].

One of the most significant distinctive features of *Brucella* organisms is the smaller genome sizes (3.1–3.4 Mb) as compared with their closest *Ochrobactrum* relatives (4.7–8.3 Mb), a phenomenon linked to their different lifestyles. Despite being acknowledged [9], Hördt et al. did not consider the qualitative and quantitative significance of the ~1.6–4.9 Mb genome size variation (Table 1 and Figure 2), corresponding to the ~950–3000 gene differences between *O. anthropi* or O. *intermedium* (the closest *Brucella* relatives) and the *Brucella* species. Some of these differences are associated with the presence of variable numbers of plasmids or chromides in the *Ochrobactrum* species [20], while in *Brucella,* the genome size and absence of plasmids is a constant trait (Table 1 and Figure 2), all characteristics of great relevance (see below). Similarly unnoticed is the biological meaning of the ~170 *Brucella* proteins whose genes are not found in the *Ochrobactrum* genomes, 40% clustering into 15 genomic sections, and the ~249 genes in 13 genomic regions unique to *Brucella* [22,23]; also critical are the idiosyncratic insertion sequences (e.g., IS711) and genomic islands with a different evolutionary history, including those encoding one Type IV secretion system and the genes involved in synthesizing the lipopolysaccharide (LPS) O-chain. It is worth noting that both are involved in virulence and the intracellular lifestyle of *Brucella* organisms [23,35,46,49,50], while the two Type IV secretion systems found in *Ochrobactrum* seem devoted to plasmid conjugation with a different evolutionary history [68].

As expected, differences in metabolic pathways and physiological processes are also vast. For example, based on genomic predictions (see BIOCYC Summary.docx in Table S1), *B. abortus* 2308 and *O. anthropi* ATCC49188 display 254 and 313 metabolic pathways, respectively, of which 35 pathways are unique to the former, and 94 pathways are unique to the latter. The differences are even more conspicuous when the reactions involved are considered (214 versus 442 are unique to each of those two strains, respectively). The differences in numbers of transport reactions, 47 for *B. abortus* 2308 and 111 for *O. anthropi* ATCC49188, are also significant and, consistent with this, in cell envelope properties, a structure critical to understanding the ecology of any bacterium. Whereas the *Ochrobactrum* species keep the selective permeability barrier necessary to thrive in an open environment, the *Brucella* species have become sensitive to hydrophobic permeants [36], an easily observable and very meaningful phenotype. All these differences show that phenotypic differences are not restricted to autapomorphies [9] and, as shown below, there are many others.

In organisms that display large long-term effective population sizes, pangenomes arise by frequently acquiring and exchanging genes for adaptation to new niches. In general, soil environments promote the expansion of bacterial pangenome size, while host-associated habitats lead to its reduction, mainly in intracellular organisms [32]. Those bacteria with large pangenomes commonly display a relatively larger number of rRNA genes and genes for broader and diverse metabolic alternatives and the molecular machinery for exchanging exogenous genes through accessory genetic elements [32]. In contrast, bacteria with smaller pangenomes have fewer rRNA genes, fewer genes coding for metabolic routes, and a reduced capacity to exchange genes. Consequently, these divergent pangenomes have been depicted as open and closed pangenomes, respectively. Pangenome sizes influence the phylogenetic history in a given bacterial group [69], and the pangenome/core genome ratio at the genus level is more pronounced than at the species level [32].

During the evolutionary history of *Brucella,* plasmids and prophages were present, and cryptic sequences of these elements have remained in the genome. An investigation of 600 strains of all classical *Brucella* species and biovars found no free plasmids [28], and the prophage sequences detected in the hundreds of genomes of strains of *Brucella* species in data banks have shown widely different origins. In at least the classical *Brucella*, most of the prophages are defective remnants and show an uneven distribution among species [31]. Upon artificial induction, only the BiPBO1 temperate phage has been recovered from *Brucella,* but, so far, it is unique to *Brucella inopinata* BO1, and it is inactive in *Ochrobactrum* [31]. As expected, the absence of recombinational events and the isolated lifestyle of *Brucella* organisms have generated a genome that, from the evolutionary perspective, acts as a close pangenome. The gentle slope of the pangenome/core genome ratio curve for the *Brucella* members on a logarithmic scale shows that the genus’s increase in genetic diversity per sequenced genome added is low (Figure 2). Accordingly, the estimated core genome size for the genus is ~1000 genes with a pangenome size close to ~11,000 genes, a figure that agrees with several studies [20,22,23,24,25,26,27]. Therefore, the dispersion of the *Brucella* species in trees constructed using different phylogenetic strategies displays good correspondence. All this evidence suggests that at some point, the *Brucella* ancestor, with a larger genome than the extant species, lost its capacity for gene exchange and dispersed in different (hosts) groups that diversified by losing and degrading non-essential genes at a different rate [23,24]. At the same time, translocation of genes within the closed genomes through the concourse of insertion sequences and mutations favored the genetic drift and *Brucella* speciation [23,24].

In contrast with *Brucella*, the *Ochrobactrum* species possess a variable number of plasmids and lysogenic phages that promote frequent conjugation and transformation events. Some of these plasmids are very large (~1.35 Mb) and have different functions for survival in open environments [23,25]. The temperate phages are easily induced, and some strains release phage particles even under non-induced conditions. None of the *Ochrobactrum* phages described have activity against *Brucella* species [29]. In addition, the *Ochrobactrum* organisms contain from 4 to 12 rRNA operons and a broad spectrum of metabolic alternatives [22,37]. Consequently, *Ochrobactrum* organisms display the characteristic wide-open pangenome of soil bacteria subjected to continuous scattering and genomic diversification [22,23]. In the *Ochrobactrum* genus, the genetic repertoire is much more extensive than the gene content of the individual strains and species, and a significant number of genes per additional sequenced genome are continuously added, as shown by the pronounced pangenome/core genome curve slope (Figure 2). As expected, the estimated core genome for the *Ochrobactrum* genus corresponds just to ~75 genes, while the complete pangenome is, up to now, close to 74,000 genes, that is, ~1000 times larger than the core genome [22]. Therefore, predicting the actual size and content of the *Ochrobactrum* pangenome is, in practical terms, unfeasible because the number of genes is constantly climbing due to numerous recombination and shuffling events. As a result, correspondence between trees is not straightforward in *Ochrobactrum* since, as discussed in Section 2, the phylogenetic dispersion and branching of the species depend on the broad gene pool used to construct the trees. These features set a profound difference with *Brucella* and make cladistic comparisons with the close pangenomes of the latter subjective choices and any subsequent conclusion on the homogeneity of these bacteria untenable. Significantly, these vast multidimensional differences were already reflected in the original definition of the *Ochrobactrum* genus. In addition to the broad and noticeable phenotypic differences, Holmes et al. [6] noted a low degree of hybridization between the *Brucella* and the closest *Ochrobactrum* species total DNAs (~20–30%), meaningfully higher between the latter and DNA of genera of soil bacteria such as *Phyllobacterium* and *Agrobacterium*.

### 3.3. The Significant Differences between Intracellular Pathogens and Opportunistic Free-Living Soil Bacteria in Evolutionary Paths and Population Structures

Soil and the intracellular milieu of animal cells are utterly different environments. As a rule, free-living Alphaproteobacteria display high genetic flexibility through large flexible open genomes and accessory genetic elements that promote genetic exchange, allowing a rapid adaptation to the sudden local variations in highly diverse ecological niches of soil. In contrast, intracellular Alphaproteobacteria usually possess close smaller genomes, cryptic plasmids, or no plasmids [21,70,71]. Accordingly, while soil bacteria arrange in reticulate evolutionary units following a sympatric evolutionary strategy, the eukaryotic cell-associated bacteria assemble as clonal evolutionary units following an allopatric strategy [32,33,34].

*Ochrobactrum* organisms follow a sympatric speciation type, with active gene flow commensurate with their open pangenome. This phenomenon reflects their natural niche, soil, and plants’ roots [44], where the selective forces include an extensive collection of bactericidal substances and organic molecules [71]. Accordingly, *Ochrobactrum* organisms are commonly highly resistant to antibiotics [47,56], capable of degrading phenolic compounds [72], petroleum wastes, and an extensive collection of xenobiotics [73,74,75,76], among many other substances, and produce toxic metal-adsorbing exopolysaccharides [42], abilities complemented by their effective outer membrane permeability barrier and an array of ancillary pumps, both essential for living in open environments [25,36].

It has been long established that cell-associated bacteria share a common ancestry with free-living bacteria [77,78], and *Ochrobactrum* and *Brucella* organisms exemplify this [5,7]. However, the genomic analysis of the *Brucella* lineage reveals an evolutionary jump in a quick burst (punctuated evolution [79]) which probably occurred during the adaptation to an intracellular lifestyle. Bacteria that originated from this event rapidly split from the *Ochrobactrum/Brucella* common ancestor following an allopatric type of speciation in an isolated environment that precluded significant horizontal gene flow and led to a close pangenome. The natural niche of all well-studied *Brucella* organisms is the intracellular milieu of animal cells and, although they can be temporarily isolated from contaminated materials, have never been found to multiply in open environments, even in organic substrates, and are seldom in contact with other bacteria when they are metabolically active [48,50,52,80]. Consequently, the selective forces exerted over *Brucella* organisms are determined by the relatively stable host environments, including inimical natural host defenses, adaptive immunity effectors, and availability of nutrients within cells [35,48,52,81,82]. Consistent with these properties, they do not have the machinery to achieve the complex metabolic processes performed by *Ochrobactrum* organisms [38,39,40,41] and are predicted to have marked metabolic pathway reductions and acquired idiosyncratic ones (see Section 3.2). Significantly, the cell envelope of *Brucella* has evolved in the opposite direction of the *Ochrobacrum* cell envelope (see below). The minor modifications in core LPS sugars cause a profound change in selective permeability and innate immunity recognition [35,46,49,83,84] (see the previous paragraph and below). Similar to the different roles accomplished by the Type IV secretion systems in the *Brucella* and *Ochrobactrum* organisms, these are good examples of exaptation and divergent evolution and not of similarities.

As discussed in Section 2, employing ecological considerations is a critical taxonomical principle, and when pathogenicity is correctly analyzed, the ability to invade cells and thrive within an intracellular niche is a refined and multidimensional ecological adaptation. Following this, it is clear why sympatric *Ochrobactrum* organisms and allopatric *Brucella* organisms represent two markedly different groups corresponding to two separate genera.

### 3.4. Pathogenicity, “Risk Groups”, and Taxonomy

Like many other saprophytes and commensals living in soil environments [85], some *Ochrobactrum* strains can fortuitously infect and cause disease in medically compromised human hosts through catheters or other medical devices [6,22,25,44,47,51,56]. In contrast, *Brucella* organisms are primary intracellular pathogens that are capable of infecting a variety of healthy vertebrates through mucosae, always cause disease, are strictly linked to their hosts for perpetuation in nature, and can multiply extensively (up to 10^11^ bacteria per g) in some tissues and spread rapidly among individuals of the same or different host species and also from mother to offspring [46,48,52,53]. Indeed, this explains the exceedingly high contagiousness of *Brucella.* Therefore, claiming that “the difference between *Brucella* and *Ochrobactrum* regarding their risk-group assignment could hardly be used as an argument against their inclusion in the same genus” [9] is mistaken since there is a marked conceptual difference between “risk group” and pathogenesis. The above-summarized differences manifest different infection dynamics and, hence, profound biological differences that need to be reflected in a truthful taxonomy, as explained below.

The opportunistic *Ochrobactrum* species induce an acute proinflammatory pyogenic response triggered by multiple innate immune mechanisms [35,51,86], and the “virulence” factors proposed [22,56] are, in specific terms, merely accidental. *Ochrobactrum* “virulence” has been assigned to antibiotic efflux pumps, the type IV secretion systems, and the presence of LPS genes [22,56]. However, antibiotic efflux pumps are not, *sensu stricto,* virulence factors. Moreover, the *Ochrobactrum* type IV secretion systems seem devoted to conjugation and not virulence (see above). Likewise, the LPS (which displays endotoxicity, in contrast to the LPS of the *Brucella*) is characteristic of all Gram-negative bacteria and not a specific trait of pathogens. No genes for any of the known widespread true bacterial virulence-associated factors are present in the 130 genomes of *Ochrobactrum* species analyzed [56]. Clearly, as with any other opportunistic microorganism, *Ochrobactrum* human infections do not depend on the bacterium’s intrinsic “virulence” properties but on the host’s immune status and iatrogenic causes [47]. Indeed, human infections described as occurring in immunocompetent hosts [47] have corresponded to a case of septic shock that followed parental administration of a solution heavily contaminated with *O. anthropi* [87] and to a patient that had undergone multiple invasive procedures, including urethral catheterization [88]. After a few days or even hours in animal and cell models, their rapid elimination highlights this absence of real pathogenicity [35].

In contrast to *Ochrobactrum*, the *Brucella* organisms display furtive behavior manifested in low proinflammatory responses, prolonged incubation times, and long-lasting infections [35,46,48,81,89]. This furtive behavior is related to many significant modifications in the so-called pathogen-associated molecular patterns of surface structures that, in contrast, remain unmodified in *Ochrobactrum* [35]. The list of differences is extensive and includes specific interactions of these two bacterial groups with phagocytes and cell receptors, complement, and other innate and adaptive immunity factors [81]. For example, the subtle structural differences of *Ochrobactrum* and *Brucella* LPS core oligosaccharide explain why the latter displays a comparatively reduced interaction with MD2, the TLR4 LPS co-receptor critical in the immune response [35,49,81,83]. An essential set of meaningful differences includes the sophisticated machinery for intracellular trafficking and replication inside cells carried by *Brucella* organisms, totally absent in *Ochrobactrum* [35,46]. These and other molecular differences eventually display multidimensional emergent heuristic attributes that reflect the deep understanding of living organisms’ essence, shaping them in space and time.

In summary, the argument that *Brucella* and *Ochrobactrum* are similar because “*Ochrobactrum* species are also known from clinical specimens, including its type species” [9] is incomplete because it does not include the complexities of the pathogenicity mechanisms (“virulence factors”). Similarly, it does not consider the fact that hosts display a wide variety of environments, rather than being a global environment, and that the barriers preventing infections are affected by the host immune status and medical manipulations. As stressed above, ecological considerations play a key role in genus definitions. From an ecological and microbiological perspective, bacterial pathogenicity is a complex phenomenon that cannot be treated in taxonomy either generally (“clinical specimens”) or as a unidimensional low-to-high quantitative property (“risk groups”) [9].

## 4. The Practical Arguments Derived from Pathogenicity and Virulence

Since the description of the genus *Brucella* by Meyer and Shaw 100 years ago [1], scientists have known that members of this genus were dangerous intracellular pathogens of animals and humans, causing severe human suffering and economic losses in the livestock industry worldwide. As said, minimal standards for inclusion in the genus stressed pathogenicity [2], and new members, keeping their zoonotic pathogen potential, have been incorporated into the genus in the last two decades [1]. Medical, microbiology, and bacteriology textbooks and manuals of bacterial classification such as the Prokaryotes and Bergey’s consistently define, as a chief characteristic, the solid pathogenic nature of *Brucella* organisms, describing how to recognize species, diagnose, prevent, and treat an infection [52,90]; however, by no means in the case of *Ochrobactrum* species. From a practical perspective, including soil environmental bacteria in the *Brucella* genus is nontrivial; it causes difficulties by confusing veterinarians, medical doctors, epidemiologists, scientists, and official authorities devoted to the treatment, surveillance, and control of brucellosis. These conundrums are particularly relevant in low- and middle-income countries where *Brucella* species remain endemic and sophisticated diagnostic tools are scarce.

### 4.1. Animal Brucellosis and Ochrobactrum

In addition to its zoonotic character, brucellosis is an economically relevant disease of important domestic animals, including bovines, caprines, ovines, swine, canines, yacks, water buffaloes, camels, reindeer, and others [52,91]. Therefore, the correct identification of *Brucella* is essential for monitoring the disease in livestock. Countries expend millions of dollars on brucellosis control programs to reach brucellosis-free status, significantly impacting international trade. *Ochrobactrum* species are ubiquitous in soil and water, and they are common contaminants in bacteriological cultures, bovine embryos, bovine semen, soil-cattle farms, food animal products, as well as bovine, ovine, and swine tissues [54,92,93,94,95,96,97]. Consequently, renaming *Ochrobactrum* organisms as *Brucella* would bring confusion to those countries that have eradicated brucellosis from livestock. For example, reporting the isolation of “Brucella intermedium” (*Ochrobactrum intermedium*) on *Brucella*-selective agar after necropsy of imported cattle in Japan [97] would immediately catch the attention of health authorities, causing unnecessary troubles. Likewise, in Bosnia and Germany, *Ochrobactrum* organisms have been isolated from tissues after necropsy of animals suspected to have brucellosis [54]. Undeniably, using the same genus name for ubiquitous soil bacteria and dangerous pathogenic organisms increases the chances of committing mistakes in vaccination, testing, and slaughtering during the execution of programs devoted to controlling and eradicating the disease from livestock, thus, causing economic distress and health problems.

### 4.2. Human Brucellosis versus Ochrobactrum Infections

Except for sporadic *Brucella canis* infections and the rare cases caused by non-classical atypical *Brucella* BO strains (only two cases reported), all other *Brucella* human infections (regardless of the *Brucella* species) can be diagnosed by straightforward, simple, fast, and inexpensive serological tests [55]. This characteristic rests in the shared surface LPS epitopes of all smooth *Brucella* species causing disease [55]. These serological assays can also be used to follow the course of brucellosis after antibiotic treatment and assess the prevalence and incidence of human and animal brucellosis [55,58,98]. Due to the antigenic surface differences among *Ochrobactrum* organisms, the serological tests are not practical and, in all respects, absent. Consequently, the diagnosis, identification, and treatment of ochrobacteriosis follow entirely different strategies [47]. Moreover, safe and effective vaccines were developed many years ago for the prophylaxis of animal brucellosis [62], limiting zoonotic infections as a key additional benefit [63]. In contrast, vaccines neither exist nor are necessary against opportunistic *Ochrobactrum*. Thus, including *Ochrobactrum* organisms in the genus *Brucella,* rather than helping, creates confusion in an already complicated diagnosis, epidemiological surveys, prevention, and treatment of human brucellosis.

The lack of plasmids and confined environment of *Brucella* species, accentuated by the absence of significant human-to-human transmission, relates to their stable susceptibility to antibiotics used in the best regimes [59]. Tetracycline and aminoglycoside sensitivity has remained constant throughout time, even in isolates recovered from relapsed patients [58], and only a low degree of resistance to rifampin has been detected in vitro in a few isolates [99,100]. The stable sensitivity of antibiotics of the classical *Brucella* species can be extended to all the known extant members of the genus, as revealed by whole-genome comparisons among the different species. Thus, independently of the *Brucella* species causing the disease, medical doctors can apply, with confidence, effective and well-defined antibiotic therapy protocols [101]. The treatment is the same even in those few infections caused by non-classical *Brucella* species that do not display the usual LPS structure, and the identification is performed by other means (e.g., PCR or MALDI-TOF MS) [60]. In contrast, *Ochrobactrum* species show broad antibiotic resistance and can develop de novo antibiotic resistance [47,56]. Consequently, testing antibiotic susceptibility in ochrobacteriosis is mandatory [47,61]. In addition, it is necessary to understand that, in ochrobacteriosis, the antibiotics generally used are a monotherapy of ciprofloxacin, trimethoprim, or sulfamethoxazole administrated for a few days or weeks [47,56], whereas the standard protocols in non-complicated brucellosis are a combined use of doxycycline plus streptomycin or rifampin for eight weeks [101].

In summary, the infectiveness, anamnesis, diagnosis, course of the disease, treatment, and recovery in brucellosis depart from opportunistic ochrobacteriosis in such a way that, from a practical standpoint, the *nomen periculosum* rule applies in all its terms [102]. The appropriateness of applying this rule is highlighted by the fact that the *Brucella* organisms are Class 3 pathogens and among the most typical causes of laboratory-acquired infections [103,104,105], which is, indeed, not the case of *Ochrobactrum* organisms. Therefore, it is necessary to keep *Brucella* and *Ochrobactrum* as two separate genera for safety and common-sense reasons.

## 5. Concluding Remarks

Cladograms do not denote everything within the context of taxonomy or evolution. In modern prokaryotic systematics, we have chosen to compare genomic sequences of the extant species because it is the most accessible and expedited manner to explore relatedness. However, a phylogenetic tree is a two-dimensional representation, not an entire taxonomic monograph, and an even less complete evolutionary history of the embodied living systems. Living organisms must not be categorized following the only rules of two-dimensional taxonomy since they are multidimensional dynamic systems that evolve in real-time according to complex spatial-temporal contingencies with a practically infinite number of genetic combinations. There are many examples in biology in which two genera closely related by cladistics are kept apart following different criteria. We can live with this if we understand the context in which taxonomy works. The evolution of pathogenesis (and many other life episodes) is not a bidimensional flat-looking circumstance but a multidimensional emergent process requiring complex and elaborated analysis. In this regard, the *Brucella* and *Ochrobactrum* case is paradigmatic. It illustrates how intracellular pathogens of animals diversify from soil bacteria into a totally distinct group, which, as demonstrated, comprises a distinct and clearly defined genus that should not be clustered with *Ochrobactrum*. As established, *Brucella* and *Ochrobactrum* genera must be maintained separately, and we urge researchers, culture collections, and databases to keep their canonical nomenclature.

Taxonomy is a valuable and practical endeavor that commonly ends with a Linnaean nomenclature. Unless we arbitrarily adopt an unnatural epistemological pan-mathematicism, any natural taxonomic scheme must incorporate analytical and typological properties (including pathogenicity). Not doing this overlooks the essence and purpose of taxonomy to serve as a valuable instrument for other disciplines. As recently underlined by scientists working with a variety of microorganisms, who have also been affected by unilateral taxonomical decisions, “Science depends on nomenclature, but nomenclature is not science,” arguing that taxonomy requires the input of a broader community of microbiologists, other scientists, and professionals working with the organisms because they are affected by such decisions [106]. Ignoring the genetic, structural, biological, medical, and practical differences between *Ochrobactrum* and *Brucella* to merge them in a single genus is not granted when the analytical and typological properties are considered and, in addition, it conveys serious risks. Moreover, one of the purposes of taxonomy is to provide names wisely linked to a set of biological characteristics uniformly shared by the living beings under a given designation.

For one hundred years, “brucellosis” has been linked to a disease caused by members of the genus *Brucella* because, regardless of the species, all induce the same syndrome. Before the genus was established, the disease received at least fifty different names, causing significant confusion in the anamnesis and diagnosis [1]. Therefore, brucellosis is a meaningful name representing a malady caused by a relatively homogeneous group of pathogenic bacteria. From the practical and historical perspective, changing the genus of *Ochrobactrum* to “*Brucella*” would alter the mainstream of microbiology laboratories, medical doctors and veterinarians, microbial collections, genome databases, microbiological and medical books, product names, and many other practical issues, mainly targeting low- and middle-income countries where the disease is endemic and/or currently emerging.

Names of microorganisms have relevant implications in medical and veterinary practices, among many other areas. If taxonomy constitutes a system of information storage, then the relevant and factual information must be easily retrievable and not confusing. Following this, what is, for example, the information retrieved from names such as “Brucella anthropi,” “Brucella pecoris,” or “Brucella lupini” (actually *Ochrobactrum anthropi*, *Ochrobactrum pecoris,* and *Ochrobactrum lupini*)? Is there a “Brucella anthropi”pathogen-specific to humans, such as *Brucella ovis* is to sheep, *Brucella canis* to dogs, or *Brucella ceti* to dolphins? Is there a new brucellosis agent of domestic livestock as “Brucella pecoris” denotes? Do yellow beans pose a risk for transmitting brucellosis and, therefore, should they be treated as a source of pathogenic selected agents? Symptomatic of the lack of practical value of merging these bacteria in a single genus, some authors simultaneously use *Brucella* and *Ochrobactrum* for the same bacterium in the publication’s title to avoid misunderstandings [107].

A phylogenetic tree is a valuable representation of an evolutionary path that species have followed and a principle to establish hypotheses. However, taxonomical ranks are not equivalent to phylogenetic analyses, as exemplified by a proper understanding of pathogenicity. Following Jacques Monod’s (1910–1976) premise, we are dealing “with living systems not with the living matter”; accordingly, we should acknowledge their multidimensional complexity.

## Figures and Tables

**Table 1:** Comparison between the *Brucella* and *Ochrobactrum* genus.

Divergent Properties	*Brucella*	*Ochrobactrum*	References
Genome Size	3.1–3.4 Mb	4.7–8.3 Mb	[20,21]
Pangenome	Closed	Open	[20,22,23,24,25,26,27]
Plasmid	No	Variable (up to 6)	[20,21,23,25,28,29]
Phylogeny	Monophyletic	Polyphyletic	[8,30]
Active Phages	No	>4	[29,31]
Lateral gene transfer	Absent	Present	[29]
Speciation type	Allopatric	Sympatric	[32,33,34]
Cell envelope permeability	Permeable to hydrophobic probes and resistant to destabilization by polycationic peptides	Impermeable to hydrophobic probes and sensitive to polycationic peptides	[35,36]
Metabolic redundancy	Low	High	[22,37]
Degradation of complex molecules	No	A large variety of such molecules	[38,39,40,41]
Removing toxic metals	No	Yes (some species/strains)	[42,43]
Capable to root nodulation	No	Yes (some species/strains)	[44,45]
Life style	Pathogen (class 3)	Saprophyte	[6,44,46]
Natural habitat	Intracellular	Soil and root plant surfaces	[24,35,44]
Transmission	Host-host interaction/animal products	Mostly iatrogenic	[47,48]
Virulence	Finely tuned	Fortuitus/opportunistic	[6,46,47]
Virulence mechanisms	Escape from the immune response/deviation of the intracellular trafficking	No true ones and virulence depending on host immune status	[35,46,49,50]
Infection dynamics	Long-lasting infection and low proinflammatory response	Acute proinflammatory/pyogenic; self-limiting in immunocompetent hosts	[46,47,51]
Animal disease	Very important	Seldom	[46,48,52,53,54]
Human health	Very important	Negligible	[22,46,47,48,52]
Diagnosis	Well-standardized serological methods	No serological tests are available or necessary	[52,55]
Treatment	WHO recommended long bi-therapy in uncomplicated cases	Based on antibiotic resistance/short monotherapy	[47,56,57]
Antibiotic resistance	Seldom and well-defined	High	[25,47,56,57,58,59,60,61]
Vaccine	Available (domestic ruminants) and critically important to control disease	Unnecessary	[62,63]
WHO/OIE/FAO regulations	Very important	Null	[57]

## Supplementary Materials

The following supporting information can be downloaded at: https://www.mdpi.com/article/10.3390/pathogens11030377/s1, Table S1: Compartive properties between Brucella abortus 2308 and Ochrobactrum anthropi ATCC49188.
