# Supplementary material for: Pathogenicity and Its Implications in Taxonomy: The Brucella and Ochrobactrum Case
Source: Pathogens. 2022 Mar 21;11(3):377. doi: 10.3390/pathogens11030377 (PMC8954888; doi:10.3390/pathogens11030377)
Supplement: Supplementary file 1 [file pathogens-11-00377-s001.zip › pathogens-1620040-supplementary.pdf]

Table S1.Compartive properties between *Brucella abortus* 2308 and *Ochrobactrum anthropi* ATCC49188. Data retrived from https://biocyc.org/comp-genomics?

Database	*B. abortus* 2308W	*O. anthropi* ATCC 49188
Genome Size (bp)	3,278,307	5,205,777
Chromosomes	2	2
Plasmids	0	4
Genes	3,035	4,859
%GC Content	57.22	56.13
Transcription Units	2,066	3,242
Pathways	254	313
Transport Reactions	47	111

Table. Shared Reactions

This table counts the reactions that are shared between pairs of organisms. The number in parentheses represent the Jaccard similarity coefficient for the reactions. Click on the first cell (Reactions Shared by Organism Pairs) to see a table listing all shared reactions. Click on a number within a cell to see a listing of those shared reactions.

**Reactions Shared by Organism Pairs**	*B. abortus* 2308	*O. anthropi* ATCC 49188
Brucella abortus 2308	1472 (1.000)	1258 (0.657)
Ochrobactrum anthropi ATCC 49188	1258 (0.657)	1700 (1.000)

Table. Unique Reactions

This table counts the reactions that are unique to each organism, i.e., are not present in any of the other organisms. Click on Unique Reactions to see a table listing all of the unique reactions. Click on a number within a cell to see a listing of the reactions unique to that organism.

**Unique Reactions in Organism**	*B. abortus* 2308	*O. anthropi* ATCC 49188
Unique Reactions	214	442

Table. Pathway Comparison by Pathway Class

This table presents statistics on the number of pathways present in each pathway class. The two largest top-level classes, Biosynthesis and Degradation/Utilization/Assimilation, are broken down further to show the distribution of pathways among their next-level subclasses. The vast majority of pathways are assigned to only a single class. However, a small number may be assigned to more than one class; such pathways would be double-counted, making the Total line in this table different from the totals in the Shared Pathways table.

To see a comparison table for all individual pathways, indicating which organisms each pathway is present in, click "Pathway Class" in the top-left corner. To see a comparison table for all pathways within a given class, click on that class name in the first column.

**Pathway Class**	*B. abortus* 2308	*O. anthropi* ATCC 49188
Biosynthesis	155	173
Amine and Polyamine Biosynthesis	3	5
Amino Acid Biosynthesis	23	28
Aminoacyl-tRNA Charging	3	2
Aromatic Compound Biosynthesis	4	5
Carbohydrate Biosynthesis	13	11
Cell Structure Biosynthesis	5	5

Cofactor, Carrier, and Vitamin Biosynthesis	49	59
Fatty Acid and Lipid Biosynthesis	17	16
Metabolic Regulator Biosynthesis	2	4
Nucleoside and Nucleotide Biosynthesis	14	18
Other Biosynthesis	1	0
Polyprenyl Biosynthesis	4	1
Secondary Metabolite Biosynthesis	3	4
Storage Compound Biosynthesis	0	0
Tetrapyrrole Biosynthesis	3	5
Generation of Precursor Metabolites and Energy	18	27
Metabolic Clusters	5	6
Bioluminescence	0	0
Detoxification	2	6
Transport	0	0
Macromolecule Modification	10	8
Activation/Inactivation/Interconversion	2	2
Degradation/Utilization/Assimilation	72	112
Alcohol Degradation	3	4
Aldehyde Degradation	0	1
Amine and Polyamine Degradation	4	9
Amino Acid Degradation	18	24
Aromatic Compound Degradation	3	3
C1 Compound Utilization and Assimilation	2	5
Carbohydrate Degradation	4	8
Carboxylate Degradation	10	13
Chlorinated Compound Degradation	0	0
Cofactor, Prosthetic Group, Electron Carrier Degradation	0	0
Degradation/Utilization/Assimilation - Other	1	1
Fatty Acid and Lipid Degradation	3	3
Hormone Degradation	0	0
Inorganic Nutrient Metabolism	8	20
Nucleoside and Nucleotide Degradation	7	9
Polymeric Compound Degradation	0	1
Protein Degradation	0	0
Secondary Metabolite Degradation	7	9
Glycan Pathways	2	0
Signal transduction pathways	0	0
Total	240	306

Table. Shared Pathways

This table counts the pathways that are shared between pairs of organisms.The number in parentheses is for the pairwise pathways comparison between two organisms - the Jaccard similarity coefficient for the pathways. Click on the first cell (Pathways Shared by Organism Pairs) to see a table listing all shared pathways. Click on a number within a cell to see a listing of those shared pathways.

**Pathways Shared by Organism Pairs**	*B. abortus* 2308	*O. anthropi* ATCC 49188
Brucella abortus 2308	224 (1.000)	189 (0.594)
Ochrobactrum anthropi ATCC 49188	189 (0.594)	283 (1.000)

Table. Unique Pathways

This table counts the pathways that are unique to each organism, i.e., are not present in any of the other organisms. Click on Unique Pathways to see a table listing all of the unique pathways. Click on a number within a cell to see a listing of the pathways unique to that organism.

**Unique Pathways in Organism**	*B. abortus* 2308	*O. anthropi* ATCC 49188
Unique Pathways	35	94
